# Panel of serum biomarkers for differential diagnosis of idiopathic interstitial lung disease and interstitial lung disease-secondary to systemic autoimmune rheumatic disease

**DOI:** 10.1371/journal.pone.0311357

**Published:** 2024-10-03

**Authors:** Miriana d’Alessandro, Paolo Cameli, Caroline V. Cotton, Janine A. Lamb, Laura Bergantini, Sara Gangi, Sarah Sugden, Lisa G. Spencer, Bruno Frediani, Robert P. New, Hector Chinoy, Elena Bargagli, Edoardo Conticini

**Affiliations:** 1 Department of Medical and Surgical Sciences & Neurosciences, Respiratory Diseases Unit, University of Siena, Siena, Italy; 2 Department of Rheumatology, Liverpool University Hospitals NHS Foundation Trust, Liverpool, United Kingdom; 3 Epidemiology and Public Health Group, School of Health Sciences, University of Manchester, Manchester, United Kingdom; 4 Aintree Chest Centre, Aintree Hospital, Liverpool, United Kingdom; 5 Department of Medicine, Surgery & Neurosciences, Rheumatology Unit, University of Siena, Siena, Italy; 6 Faculty of Biology, Medicine and Health, Division of Musculoskeletal and Dermatological Sciences, The University of Manchester, Manchester, United Kingdom; 7 Department of Rheumatology, Salford Royal Hospital, Northern Care Alliance NHS Foundation Trust, Manchester Academic Health Science Centre, Salford, United Kingdom; Nippon Medical School, JAPAN

## Abstract

**Background:**

Interstitial lung disease (ILD) may complicate the course of systemic autoimmune rheumatic disease (SARD) and diagnostic biomarkers are needed. Krebs von den Lungen-6 (KL-6), ferritin (FER) and interleukin 6 (IL-6) have been involved in the ILD development. Our study aimed to compare KL-6, FER, IL-6 and soluble mesothelin-related peptide (SMRP) concentrations in a cohort of idiopathic and SARD-ILD.

**Methods:**

3169 patients were enrolled in the “UK Biomarkers in Interstitial Lung Disease (UK-BILD) Study”. We selected patients affected by SARD-ILD and idiopathic ILD (usual interstitial pneumonia-idiopathic pulmonary fibrosis and fibrotic non-specific interstitial pneumonia). Serum marker concentrations were measured through chemiluminescent assays (Fujirebio Europe, Ghent, Belgium).

**Results:**

1013 patients were selected for the study: 520 (51.3%) had idiopathic ILD and 493 (48.7%) SARD-ILD. Idiopathic ILD patients displayed higher KL-6 values than SARD-ILD (p = 0.0002). FER and SMRP, though within normal ranges, were significantly higher in idiopathic ILD (p<0.0001). Logistic regression showed good sensitivity (69.4%) and specificity (80.4%) selecting the variables FER and KL-6 concentrations, age and gender-male correlated with a diagnosis of idiopathic ILD.

**Conclusion:**

Our study showed the excellent diagnostic value of KL-6 for detecting ILD, which irrespective of the final diagnosis and extent of disease, is always elevated and is a reliable biomarker of lung fibrosis in various diseases, ranging from idiopathic to autoimmune forms. Our study proposed an ILD differentiation model including clinical background. In this context, combination of serum markers and clinical data, as seen in our cohort, may lead to a further improvement in diagnostic accuracy for ILD.

## Introduction

Interstitial lung disease (ILD) may complicate the course of systemic autoimmune rheumatic disease (SARD) [1,2], and is one of the leading causes of morbidity and mortality [3,4]. No definite diagnostic work-up has yet been validated for these patients, nor does robust evidence exist for optimal management, as SARD-ILD not always responds to conventional immunosuppressants, which are the mainstay of therapy for SARD. Diagnosis and management of SARD-ILD requires a comprehensive, multidisciplinary, individualized approach that relies mainly on pulmonary function tests (PFT), high-resolution computed tomography (HRCT) of the chest and sometimes also lung biopsy [5]. A multidisciplinary assessment is highly recommended by international guidelines for the diagnostic pathway of ILD, including at least pulmonologists, radiologists, rheumatologists, in order to optimise the diagnostic accuracy and guarantee the earliest and more proper therapeutic proposal. Despite this recommendation, a significant percentage of ILD patients still receives a “working diagnosis”, since clinical symptoms and immunological assessment may not always be sufficient for a confident diagnosis and potentially invasive samplings (such as cryobiopsy or lung surgical biopsy) may not be suitable due to the frailty of clinical conditions [6]. Non-invasive biomarkers for early detection of lung involvement and its severity are badly needed.

Krebs von den Lungen-6 (KL-6) is a high-molecular-weight glycoprotein mainly expressed on proliferating, regenerating and injured type II alveolar epithelial cells (AECs) [7]. It has been suggested as a mainly prognostic serum marker of fibrosis in ILD patients, including those with idiopathic pulmonary fibrosis (IPF) [8,9] and hypersensitivity pneumonitis (HP) [10,11], as well as in SARD-ILD patients, including those with anti-synthetase syndrome (ASS) [12], dermatomyositis (DM) [13,14], systemic sclerosis (SSc) [15–18], primary Sjögren’s syndrome (pSS) [19,20], rheumatoid arthritis (RA) [21,22] and ANCA-associated vasculitis [23]. KL-6 concentrations seem to have a positive correlation with the degree of lung impairment detectable by HRCT and a negative correlation with forced vital capacity (FVC) and diffusing capacity of the lungs for carbon monoxide (DLCO) [24]. Since this correlation reflects the severity of SARD-ILD, it can be useful to select patients who could benefit from HRCT and PFT and spare others excessive exposure to radiation and unnecessary procedures, while reducing healthcare costs. Interestingly, in patients with confirmed ILD, KL-6 may decrease during remission of inflammatory activity, but usually remains above normal values.

As far as other serum biomarkers are concerned, ferritin (FER) is a key protein of iron metabolism capable of sequestering large amounts of iron, and thus serves the dual function of iron detoxification and iron storage; it seems to be an important regulator of the immune system, playing a central role in autoimmune diseases [25]. A growing body of data shows that serum FER is correlated with disease activity and poor prognosis in anti-MDA5-positive DM-ILD patients [26,27], with reported cut-off values that vary from 500 to 1500 ng/ml [28]. Conversely, no data exists on serum FER in other forms of SARD. Interleukin 6 (IL-6) is a pleiotropic cytokine involved in the physiology of virtually every organ system. Controlling IL-6 activity is potentially an effective approach in the treatment of various autoimmune and inflammatory diseases. On the other hand, like calretinin, a well-known marker correlated with IPF severity, soluble mesothelin-related peptide (SMRP) is a surface marker of mesothelial cells, such as pleural mesothelial cells (PMC). No data is available on the role of SMRP in SARD-ILD patients. Nevertheless, recent evidence highlights the role of mesothelin (MSLN which binds cancer antigen CA-125 also known as MUC16) in pulmonary fibrosis, suggesting that MSLN is involved in cell adhesion [29]. The literature reports a role of MUC16 in the development and progression of IPF through the TGF-β1 canonical pathway. The above evidence suggests that FER, KL-6, SMRP and IL-6 may provide a serum biomarker profile that can distinguish the progression of fibrotic damage due to inflammatory activity in SARD-ILD, making it possible to optimize therapeutic management with immunosuppressants and/or antifibrotics.

Here we explore the landscape of serum biomarkers in idiopathic and SARD-ILD in a large cohort of patients from the UK Biomarkers in Interstitial Lung Disease (UK-BILD) Study. The primary endpoint of our study was to assess serum concentrations of IL-6, SMRP, KL-6 and FER in a large cohort of idiopathic or non-idiopathic ILD patients. Secondary endpoints were: to assess whether these biomarkers may be considered specific for SARD-ILD as distinct from idiopathic ILD; to evaluate the association with clinical and imaging findings; and to construct a panel for differential diagnosis.

## Methods

Patients included in the “UK Biomarkers in Interstitial Lung Disease (UK-BILD) Study” were retrospectively enrolled from 39 UK recruitment centres between 07^th^ January 2015 and 07^th^ December 2018. The UK-BILD cohort recruited 3169 in which patients must have HRCT-proven ILD, and their investigations must have included “routine” serology. All recruiting clinicians completed a two-page clinical proforma documenting the following data: age, gender, ethnicity, smoking history, diagnosis of SARD-ILD and idiopathic ILD, SARD signs (including Raynaud, arthralgia/arthritis, sclerodactyly, calcinosis, elevated CK, mechanic’s hands, myalgia, periungual erythema, telangiectasia), ILD signs (including digital clubbing, inspiratory crackles, pulmonary hypertension features) and recruiting centre information.

The SARD-ILD group included patients with a diagnosis of pSS, RA, systemic lupus erythematous (SLE), mixed connective tissue disease (MCTD), polymyositis (PM), dermatomyositis (DM), undifferentiated connective tissue disease (UCTD), limited and diffuse SSc and unknown CTD, according to international classification criteria [30–37]. The idiopathic ILD group included patients with a diagnosis of usual interstitial pneumonia-(UIP-)IPF and fibrotic non-specific interstitial pneumonia (NSIP), diagnosed according to American Thoracic Society/European Respiratory Society (ATS/ERS) guidelines [38]. In all Centres, multidisciplinary discussion for diagnostic assessment included respiratory physicians, radiologists, rheumatologists and, in case of tissue sampling for diagnostic purposes, histopathologists, all with a specific expertise in ILD setting. The study was conducted according to the guidelines of the Declaration of Helsinki and approved for “UK Biomarkers in Interstitial Lung Disease (UK-BILD) Study”. All patients gave their written informed consent to participation in the study.

For biomarker analysis, inclusion criteria were a diagnosis of IPF, idiopathic NSIP and SARD-ILD; exclusion criteria were a diagnosis of hypersensitivity pneumonitis, sarcoidosis, asbestosis, idiopathic cryptogenic organizing pneumonia, respiratory bronchiolitis, Langerhans cell’s histiocytosis, lymphangioleiomyomatosis, desquamative interstitial pneumonia, acute interstitial pneumonia, lack of serum sample, insufficient or no clinical data, a previous diagnosis (last 5 years) of malignancy and too few patients for statistical analysis.

Serum samples were obtained from recruited patients, anonymized in an electronic database and marker concentrations were measured singly by KL-6, IL-6, FER and SMRP reagent assays (Fujirebio Europe, Ghent, Belgium). The reagents were designed for fully automated chemiluminescent enzyme immunoassay with the LUMIPULSE G System (Fujirebio Europe, Ghent, Belgium). The principle of the assay is agglutination of sialylated carbohydrate antigen with KL-6, IL-6, FER and SMRP mAbs by antigen-antibody reaction. The change in absorbance was measured to determine serum concentrations of KL-6 expressed in U/mL, IL-6 in pg/mL, FER in ng/mL and SMRP in nmol/L. Reference calibrator values were 0 and 1000 ng/mL for FER, 0, 2 and 100 nmol/L for SMRP, 0, 500 and 10000 U/mL for KL-6 and 0, 20, 400 and 1000 pg/mL for IL-6. The reference intervals for FER concentrations in the low range were 31.5–75.0 ng/mL, and in the high range 280–520 ng/mL. The reference ranges for SMRP were 1.11–1.84 nmol/L (low) and 9.39–15.65 nmol/L (high), and for KL-6 258–387 U/mL (low) and 659–988 U/mL (high). For IL-6 we used the standardized reference ranges 32.2–49.4 pg/mL (low) and 195–299 pg/mL (high) [39].

### Statistical analysis

All data is reported as mean ± standard deviation or median and interquartile range (IQR), as appropriate. The Shapiro-Wilk test was used to determine normal distribution. Multiple comparisons were assessed by non-parametric one-way ANOVA (Kruskal-Wallis test) and the Dunn test. The validity of serum marker concentrations used to distinguish SARD-ILD and idiopathic ILD patients was assessed by areas under the receiver operating characteristic (AUROC) curve. Sensitivity and specificity were calculated for cut-offs of the different variables. The Youden index (J = max [sensitivity + specificity − 1]) was used to establish the best cut-offs.

Patients were further stratified according to HRCT findings and comparative analysis of serum marker concentrations were performed within and between the following groups: the idiopathic ILD group included probable fibrotic NSIP on HRCT (IPF confirmed at multidisciplinary discussion, >65 years old, without UIP confirmation at lung biopsy), definite UIP and definite fibrotic NSIP (UIP confirmed at lung biopsy). For SARD patients, those with RA and SSc displaying a UIP pattern (SARD-UIP) were considered separately from those with NSIP (SARD-NSIP).

Machine learning analysis with variable-importance plot was performed to construct a model selecting variables to make accurate predictions. The more a model relies on a variable to make predictions, the more important it is for the model. Binomial logistic regression and ROC curve analysis were used to predict the diagnostic value of each serum marker/clinical parameter for SARD-ILD against clinical diagnosis. Supervised Principal Component Analysis using Kaiser-Guttman rule was performed in an exploratory approach to identify trends in immunological (KL-6, IL-6, SMRP, FER) and demographic (age) features by 2D representation of the multi-dimensional data set.A p-value less than 0.05 was considered statistically significant. Statistical analysis was performed with GraphPad Prism 9.3 and Jamovi software 2.3.

## Results

The total number of patients selected for the study from UK-BILD cohort was 1239. We excluded 108 (8.7%) from the study due to insufficient or unavailable demographic and clinical data, 103 (8.3%) due to malignancies, 3 (0.2%) due to inclusion body myositis, 1 due to anti-synthetase syndrome (0.08%) and 11 (0.9%) due to an unknown disease subtype. The remaining 1013 patients (median and interquartile range, 70 (61–77) years) were enrolled in the study: 520 (51.3%) had idiopathic ILD and 493 (48.7%) had been diagnosed with SARD-ILD. Their demographic, clinical and immunological data is reported in Table 1.

**Table 1:** Demographic data, including age, gender, smoking and ethnicity in idiopathic ILD and SARD-ILD groups.

	Idiopathic ILD (N = 520)	SARD-ILD (N = 493)	p value
**age**			** **
Median (IQR)	73.5 (68–79)	64 (54–72)	**<0.0001**
**Gender, n (%)**			<0.0001
Female	132.0 (25.4%)	326.0 (66.1%)	
Male	388.0 (74.6%)	167.0 (33.9%)	
**smoking history, n (%)**			<0.0001
Never smoker	167.0 (32.1%)	246.0 (49.9%)	
Former or current smoker	353.0 (67.9%)	247.0 (50.1%)	
**ARD subgroup, n (%)**			
SSc Limited		48.0 (9.7%)	
SSc Diffuse		24.0 (4.9%)	
UCTD		42.0 (8.5%)	
CTD (Unknown)		6.0 (1.2%)	
MCTD		29.0 (5.9%)	
PM		27.0 (5.5%)	
DM		26.0 (5.3%)	
Sjogren syndrome		21.0 (4.3%)	
RA		198.0 (40.2%)	
SLE		19.0 (3.9%)	
Other		53.0 (10.8%)	
**Ethnicity, n (%)**			<0.0001
Caucasian	512.0 (98.5%)	382.0 (77.5%)	
Asian	6.0 (1.2%)	44.0 (8.9%)	
African	0.0 (0.0%)	15.0 (3.0%)	
Afro-Caribbean	1.0 (0.2%)	38.0 (7.7%)	
Others–specify	0.0 (0.0%)	7.0 (1.4%)	
Mixed–specify	1.0 (0.2%)	7.0 (0.0%)	
**IPF diagnosis, n (%)**			
Not-UIP	47.0 (9.0%)		
Definite UIP by HRCT	332.0 (63.8%)		
Definite Fib NSIP on HRCT and LBx with UIP	35.0 (6.7%)		
Probable Fib NSIP on HRCT no LBx, >65 yrs old and MDT diagnosis IPF	106.0 (20.4%)		
**PULMONARY SIGNS/SYMPTOMS**			
**Clubbing, n (%)**			<0.0001
No	377.0 (72.5%)	450.0 (91.3%)	
Yes	143.0 (27.5%)	43.0 (8.7%)	
**End inspiratory crackle, n (%)**			<0.0001
No	150.0 (28.8%)	197.0 (40.0%)	
Yes	370.0 (71.2%)	296.0 (60.0%)	
Pulmonary hypertension*, n (%)			0.1510
No	508.0 (97.7%)	475.0 (96.3%)	
Yes	12.0 (2.3%)	18.0 (3.7%)	
**ARD SIGNS/SYMPTOMS:**			
**Sclerodactyly, n (%)**			<0.0001
No	519.0 (99.8%)	437.0 (88.6%)	
Yes	1.0 (0.2%)	56.0 (11.4%)	
**Calcinosis, n (%)**			<0.0001
No	519.0 (99.8%)	473.0 (95.9%)	
Yes	1.0 (0.2%)	20.0 (4.1%)	
**Raised ck, n (%)**			<0.0001
No	517.0 (99.4%)	443.0 (89.9%)	
Yes	3.0 (0.6%)	50.0 (10.1%)	
**Mechanic’s hand, n (%)**			<0.0001
No	519.0 (99.8%)	458.0 (92.9%)	
Yes	1.0 (0.2%)	35.0 (7.1%)	
**Myalgia, n (%)**			<0.0001
None	518.0 (99.6%)	429.0 (87.0%)	
Yes	2.0 (0.4%)	64.0 (13.0%)	
**Periungual erythema, n (%)**			0.0043
No	492.0 (94.6%)	440.0 (89.2%)	
Yes	28.0 (5.4%)	53.0 (10.8%)	
**Telangiectasia, n (%)**			<0.0001
No	518.0 (99.6%)	447.0 (90.7%)	
Yes	2.0 (0.4%)	46.0 (9.3%)	
**Arthralgia/arthritis, n (%)**			<0.0001
No	508.0 (97.7%)	213.0 (43.2%)	
Yes	12.0 (2.3%)	280.0 (56.8%)	
**Raynaud, n (%)**			<0.0001
No	510.0 (98.1%)	311.0 (63.1%)	
Yes	10.0 (1.9%)	182.0 (36.9%)	
**Laboratory parameters:**			
**KL-6 U/mL**			
Mean (SD)	1604.4 (1235.2)	1522.9 (1430.8)	0.0002
**FER ng/mL**			
Mean (SD)	134.3 (138.4)	87.3 (105.4)	<0.0001
**IL-6 pg/mL**			
Mean (SD)	87.8 (222.4)	99.6 (241.4)	0.4673
**SMRP nmol/L**			
Mean (SD)	1.2 (0.7)	1.1 (0.7)	<0.0001

Clinical findings including ATD subgroups, HRCT patterns and rheumatological signs. Immunological data including serum concentrations of KL-6, FER, IL-6 and SMRP in the ILD and SARD-ILD groups. Abbreviations: ILD, interstitial lung disease; SARD, autoimmune rheumatic disease; RA, rheumatoid arthritis; SLE, systemic lupus erythematous; MCTD, mixed connective tissue disease; UCTD, undifferentiated connective tissue disease; PM, polymyositis; DM, dermatomyositis; SSc, systemic sclerosis; CTD, connective tissue disease; IPF, idiopathic pulmonary fibrosis; UIP, usual interstitial pneumonia; HRCT, high resolution computed tomography; NSIP, non-specific interstitial pneumonia; MDT, multidisciplinary discussion team; LBx, lung biopsy; KL-6, krebs von den lungen-6; FER, ferritin; IL-6, interleukin-6; SMRP, soluble mesothelin-related peptide.

*: Defined as mean pulmonary arterial pressure > 20 mmHg, measured through right heart catheterization.

### Idiopathic ILD versus SARD-ILD

**Fig 1:**
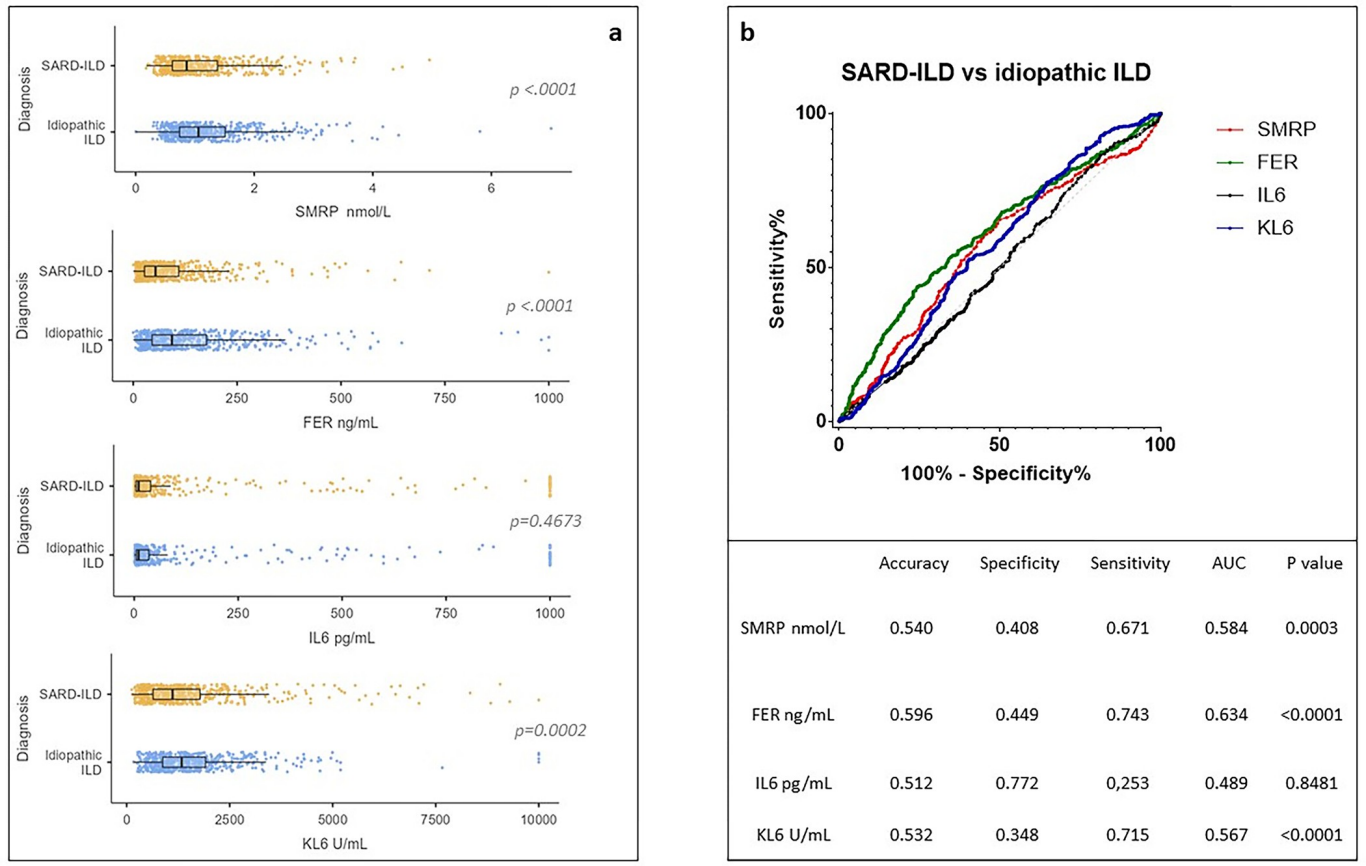
KL-6, FER, IL-6 and SMRP concentrations in SARD-ILD and idiopathic ILD groups. Comparative analysis of median concentrations of four markers in the two subgroups (1a) and ROC curve (1b) of serum biomarkers of patients with idiopathic ILD and SARD-ILD reporting specificity, sensitivity, area under the curve and diagnostic accuracy. Abbreviations: KL-6, Krebs von den Lungen-6; FER, ferritin; IL-6, interleukin-6; SMRP, soluble mesothelin-related peptide; ILD, interstitial lung diseases; SARD-ILD, systemic autoimmune rheumatic diseases associated with interstitial lung diseases.

A higher percentage of older males and former smokers (p<0.0001) was found in the idiopathic ILD group (Table 1). As expected, the two groups showed a clear discrepancy in terms of clinical features. Comparative analysis of serum biomarkers showed higher KL-6 concentrations (Fig 1A) in idiopathic ILD than in SARD-ILD patients (p = 0.0002). Although SMRP and FER concentrations were higher in idiopathic ILD than in SARD-ILD patients (p<0.0001), they remained within normal ranges. IL-6 concentrations were similar in the two groups and were in the normal range. Fig 1B shows the ROC curve to distinguish the two groups on the basis of a SMRP cut-off value of 0.88 nmol/L (sensitivity 52%, specificity 64%), a FER cut-off value of 59.15 ng/mL (sensitivity 54%, specificity 67%) and a KL-6 cut-off value of 1281 U/mL (sensitivity 59%, specificity 54%).

**Fig 2:**
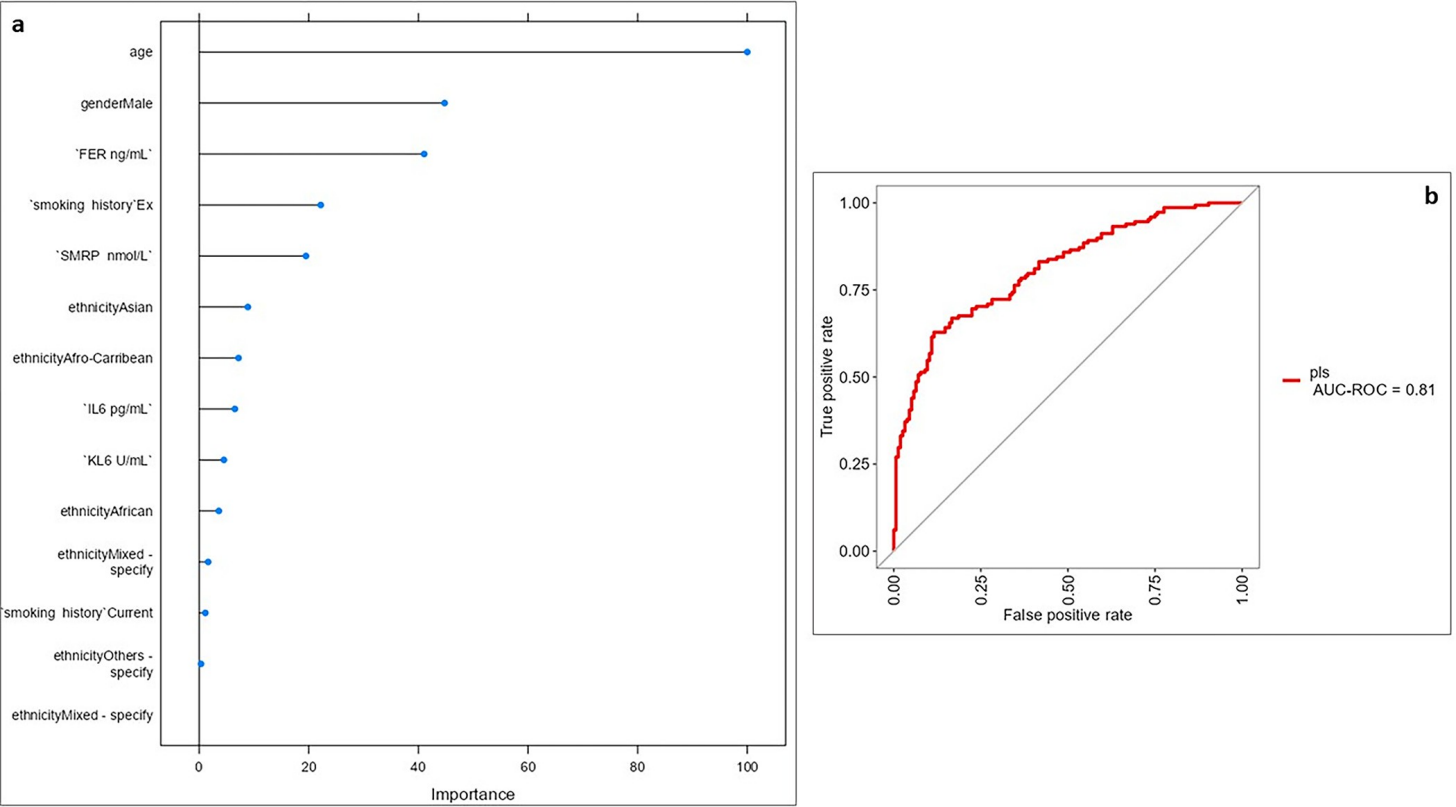
Variable-importance plot (a) selecting variables to include in the model to obtain accurate predictions: Age, gender-male, FER, SMRP, smoking history, ethnicity-Asian, IL-6, ethnicity-Afro-Caribbean and KL-6. The area under the receiver operating characteristics (AUC-ROC) curve (b) of the model was 0.81.Abbreviations: KL-6, Krebs von den Lungen-6; FER, ferritin; IL-6, interleukin-6; SMRP, soluble mesothelin-related peptide; ILD, interstitial lung diseases; SARD-ILD, systemic autoimmune rheumatic diseases associated with interstitial lung diseases.

Machine learning analysis with variable-importance plot (Fig 2) was used to select variables to include in the model to obtain accurate predictions. The more a model relies on a variable to make predictions, the more important it is for the model. The variables selected were age, gender-male, FER, SMRP, smoking history, ethnicity-Asian, IL-6, ethnicity-Afro-Caribbean and KL-6: the resulting model showed an accuracy of 0.755 (kappa 0.5087) and AUROC 0.81.

**Fig 3:**
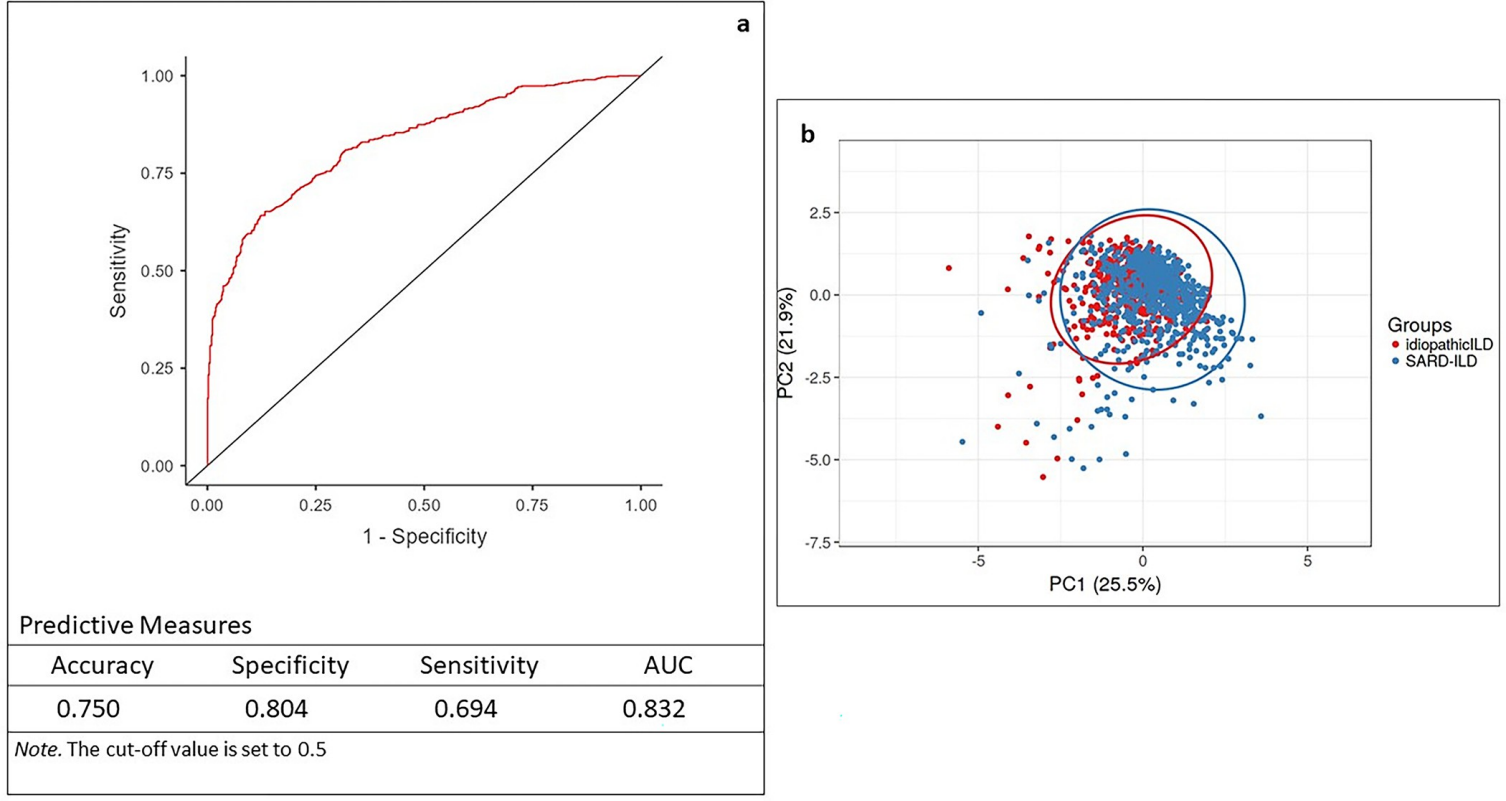
Logistic regression model (a) showed an area under the curve (AUC) of 0.832 and an accuracy of 0.75. Principal Component Analysis (b) plot showed that the idiopathic ILD and SARD-ILD groups separated on the basis of the selected variables with a total variance of 47.4%. Abbreviations: PC, principal component; ILD, interstitial lung diseases; SARD-ILD, systemic autoimmune rheumatic diseases associated with interstitial lung diseases.

Binomial logistic regression analysis (S1 Table) was performed to understand the effect of demographic (gender, age, ethnicity and smoking history) and immunological (SMRP, FER, KL-6 and IL-6) features on the diagnosis of idiopathic and SARD ILD. The variables most associated with idiopathic ILD were higher concentrations of FER (p = 0.0028) and KL-6 (p = 0.0340), age (p<0.0001) and gender-male (p<0.0001). Higher serum concentrations of FER, KL-6 and IL-6 were recorded in males than females (p<0.0001, p = 0.0014 and p = 0.0209, respectively). The variable ethnicity (Asian and Afro-Caribbean vs Caucasian) was associated with SARD-ILD (p<0.0001). The logistic regression model (Fig 3A) showed an AUROC of 0.832 with best sensitivity (69.4%) and specificity (80.4%).

The supervised Principal Component Analysis plot (Fig 3B) shows how the two groups (idiopathic ILD and SARD-ILD) separated on the basis of selected variables. The first and second components explained 47.4% of the total variance based on immunological and clinical findings showing good clustering for idiopathic ILD and SARD-ILD. The scree plot of Eigenvalues for each principal component was reported in S1 Fig.

According to HRCT stratification, serum markers were compared within and between groups and significant differences were reported in Table 2.

**Table 2:** Serum markers concentrations in groups of patients stratified according to HRCT findings: The idiopathic ILD group included probable fibrotic NSIP on HRCT (IPF confirmed at multidisciplinary discussion, >65 years old, without UIP confirmation at lung biopsy), definite UIP and definite fibrotic NSIP (UIP confirmed at lung biopsy).

Pairwise comparisons—SMRP nmol/L		Weight	P values
**S**ARD-NSIP	idiopathic probable fibrotic NSIP	47.904	0.0092
SARD-NSIP	Definite UIP	52.858	0.0026
SARD-UIP	idiopathic definite UIP	-41.369	0.0403
**Pairwise comparisons—FER ng/mL**			** **
**SARD**-NSIP	idiopathic probable fibrotic NSIP	63.024	0.0001
SARD-NSIP	DefiniteUIP	67.027	< .0001
Probable fibrotic NSIP	SARD-UIP	-70.562	< .0001
Definite UIP	SARD-UIP	-81.872	< .0001
Definite fibrotic NSIP	SARD-UIP	-40.380	0.0493
**Pairwise comparisons—IL6 pg/mL**			
SARD-NSIP	idiopathic fibrotic NSIP	5.233	0.0030
SARD-NSIP	Definite UIP	5.750	0.0007
SARD-NSIP	SARD-UIP	6.528	< .0001
idiopathic fibroticNSIP	Probable fibrotic NSIP	-4.114	0.0422
**Pairwise comparisons—KL6 U/mL**			
SARD-NSIP	SARD-UIP	-42.924	0.0291
Probable fibrotic NSIP	SARD-UIP	-48.549	0.0079
Definite UIP	SARD-UIP	-60.183	0.0003

For SARD patients, those with RA and SSc displaying a UIP pattern (SARD-UIP) were considered separately from those with NSIP (SARD-NSIP). Abbreviations: SARD, autoimmune rheumatic disease; NSIP, non-specific interstitial pneumonia; UIP, usual interstitial pneumonia; KL-6, krebs von den lungen-6; FER, ferritin; IL-6, interleukin-6; SMRP, soluble mesothelin-related peptide.

### SARD-ILD subgroup analysis

**Fig 4:**
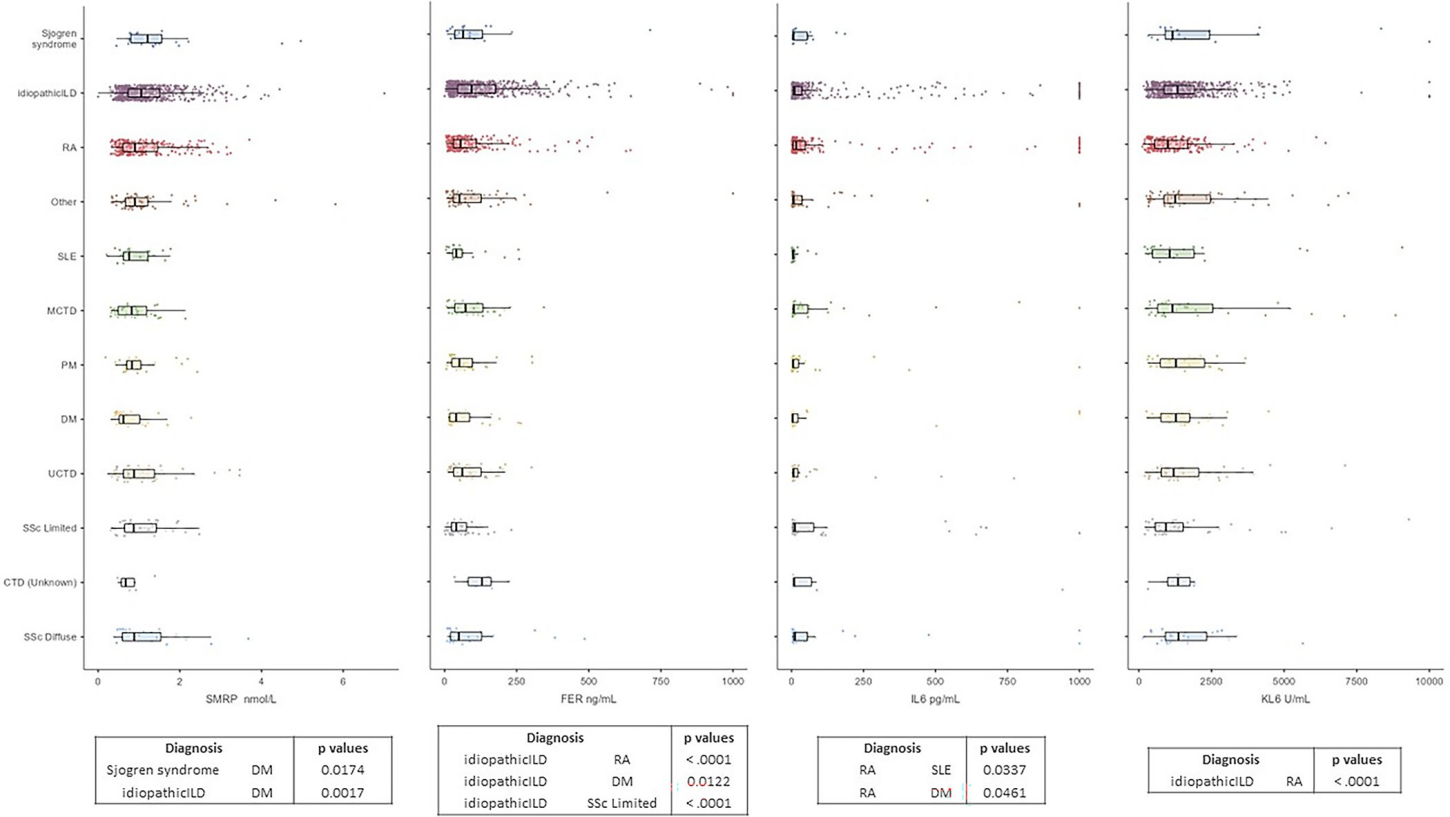
Box plots reported serum marker concentrations in idiopathic interstitial lung disease (ILD) versus SARD-ILD subgroups: Rheumatoid arthritis (RA), systemic lupus erythematous (SLE), mixed connective tissue disease (MCTD), polymyositis (PM), dermatomyositis (DM), systemic sclerosis (SSc), undifferentiated connective tissue disease (UCTD), connective tissue disease (CTD). The statistically significant differences of each serum marker concentration between the disease groups were reported in the tables below the box plots.

Patients with SARD were further subdivided according to the specific diagnosis and serum markers, and were compared within and between groups, as well as with idiopathic ILD group (Fig 4).

Finally, patients with SARD were further subdivided according to their signs and symptoms. Those who complained of Raynaud symptoms had lower serum concentrations of SMRP and FER (p<0.0001); arthralgia/arthritis was associated with lower KL-6, SMRP and FER (p = 0.0048, p = 0.0002 and p<0.0001, respectively); sclerodactyly and mechanic’s hands with lower FER (p = 0.0192 and p = 0.0364, respectively); elevated CK with lower SMRP, FER and IL-6 concentrations (p<0.0001, p = 0.0096 and p = 0.0004, respectively); myalgia with lower SMRP and FER concentrations (p = 0.0032 and p = 0.0191) and periungual erythema with lower SMRP values (p = 0.0110).

Concerning pulmonary signs, patients who showed clubbing showed higher serum concentrations of FER and KL-6 (p = 0.0347 and p = 0.0004, respectively) as well as end inspiratory crackle (p = 0.0279 and p = 0.0002, respectively).

## Discussion

Our multicentre, retrospective study is the first and largest to evaluate an extended panel of serum biomarkers in patients with different types of ILD, including SARD-ILD. We observed normal serum concentrations of IL-6, FER and SMRP in both groups, whereas KL-6 appeared above normal cut-off in most patients but was significantly higher in the idiopathic ILD group.

These findings, underlining the greater sensitivity and accuracy of KL-6 in the diagnosis of ILD, are not surprising. As early as 2000, Nakajima et al. evaluated serum KL-6 in SARD patients with and without ILD, demonstrating the potential of KL-6 as predictor of lung interstitial involvement and proposing it as a marker of disease activity [40]. Since then, other studies, mainly focusing on SSc, have shown the reliable diagnostic and prognostic value of KL-6 in SARD-ILD: KL-6 seems able to distinguish patients with and without lung involvement at an early stage and shows moderate to high correlations with lung function parameters and quantitative HRCT scores of lung interstitial involvement [41]. The specificity of KL-6 is shown by its capacity to distinguish fibrotic ILD from other types of lung involvement, such as nodular or haemorrhagic pattern in ANCA-associated vasculitis [23].

Ours is the first study to attempt a direct comparison of KL-6 in two groups of ILD. A statistically significant difference was detected: patients suffering from idiopathic ILD displayed higher levels of KL-6 than SARD-ILD patients, suggesting that this biomarker has very high specificity for idiopathic ILD and that different cut-off values are needed for other types of ILD. Likewise FER and SMRP, though within normal ranges, were significantly higher in idiopathic ILD, whereas no statistically significant difference was found for IL-6, which remained within its normal range.

These findings enabled us to build a model with an accuracy of 0.755 for differential diagnosis of idiopathic ILD and SARD-ILD based on the following variables: age, gender-male, FER, SMRP, smoking history, Asian and Afro-Caribbean ethnicity, IL-6 and KL-6. FER and KL-6 concentrations, age and gender-male predicted the diagnosis of idiopathic ILD.

Identification of biomarkers by machine learning classifiers to assist diagnose RA-ILD has been proposed by Qin et al [22]. KL-6 concentration, D-dimer, and tumor markers greatly aided RA-ILD identification. Machine learning algorithms combined with traditional biostatistical analysis could be helpful to diagnose RA-ILD patients and identify biomarkers potentially associated with the disease. Recently, Huang et al [42] proposed multiple machine learning models trained with a large number of proteins involved in the immune pathway consistently distinguished CTD-ILD from IPF in challenging cases and improved clinical decision making.

This is of paramount importance in practice, first because it may help refine and accelerate diagnostic work-up, secondly and more importantly because unnecessary treatment can be avoided and therapy can be targeted.

In order to reduce the risk of bias and to bring our analysis into line with clinical practice, where HRCT has already been performed upon referral, patients were stratified according to radiological pattern. Notably, serum concentrations of FER and SMRP were significantly higher in patients with idiopathic NSIP and UIP than in those with SARD-NSIP and UIP, respectively. At the same time, not only were serum concentrations of KL-6 higher in idiopathic NSIP and UIP patients than in those with SARD-UIP, but also in patients with SARD-NSIP than in those with SARD-UIP.

In a nutshell, elevated levels of KL-6 in a patient with suspected or even radiologically confirmed lung fibrosis are associated with a high probability of ILD. Although FER and SMRP may be in the normal ranges, their increase suggests a diagnosis of idiopathic ILD, and does not support a diagnosis of SARD-ILD. New cut-off values for FER and SMRP, specific for lung fibrosis, could make these biomarkers even more useful in clinical practice.

When we analysed patients with any form of SARD in order to refine panel sensitivity and specificity, we failed to find any statistically significant differences between subgroups, except in the case of IL-6, which was higher in rheumatoid arthritis patients. This is unsurprising given the pivotal role of this cytokine in the pathogenesis of RA.

On the other hand, interesting insights emerged from clinical findings: patients presenting with signs and symptoms of advanced lung fibrosis (end inspiratory crackles and digital clubbing) had higher serum levels of FER and KL-6, while lower levels of FER, KL-6 and SMRP were recorded in those with extra-pulmonary signs (i.e. arthralgia/arthritis, sclerodactyly, elevated CK, myalgia, periungual erythema, mechanic’s hands).

Our study has several limitations: 1) lack of any information about disease activity of the concomitant rheumatic disorders at the time of serum collection; 2) since no lung function data was recorded in UK-BILD, we were unable to compare functional data with serological and imaging findings; 3) autoimmune profile was not included in the proforma: these aspects may have been relevant for stratifying DM subtypes (namely dermatomyositis with anti-MDA5, in which FER is increased) and to refine the diagnosis of many idiopathic NSIP potentially hiding antisynthetase syndrome [43]; such an aspect is worthwhile to be further indagated in upcoming studies; 4) since we lacked a control group of SARD without ILD, we were unable to assess the specificity of FER, SMRP and IL-6.

In conclusion, our study showed the good diagnostic value of KL-6 for detecting ILD, which irrespective of the final diagnosis and extent of disease, seems to be a reliable biomarker of lung fibrosis in various diseases, ranging from idiopathic to autoimmune forms. We confirmed that KL-6 values above 500 U/mL seem to support a diagnosis of ILD in SARD patients (i.e. SSc or IIM-ILD) prior or complementary to HCRT. We also found that assay of serum concentrations of KL-6, combined with FER and SMRP, is useful for differential diagnosis: serum cut-off values of KL-6, FER and SMRP, the latter two within normal values, were validated for differential diagnosis of idiopathic ILD and SARD-ILD. To the best of our knowledge, this is the first time that FER has been thoroughly investigated in patients with lung fibrosis, other than dermatomyositis with anti-MDA5. At the same time, there was no previous data on the role of SMRP in ILD patients and ours is the first study to report higher serum concentrations of SMRP in idiopathic ILD than in SARD-ILD patients, suggesting its potential for differential diagnosis. In this context, combination of serum markers and clinical data, as seen in our cohort, may lead to a further improvement in diagnostic accuracy for ILD.

## Supporting information

S1 Table Binomial logistic regression was performed to understand the effect of demographic (gender, age, ethnicity and smoking history) and immunological (SMRP, FER, KL-6 and IL-6) features on the diagnosis of idiopathic ILD and SARD-ILD. The statistically significant variables was marked in bold (p values columns).
(DOCX)

S1 Fig The scree plot method for Kaiser-Guttman’s rule to conduct supervised Principal Component Analysis in an exploratory approach for identifying trends in immunological (KL-6, IL-6, SMRP, FER) and demographic (age) features by 2D representation of the multi-dimensional data set. (DOCX)

## Abbreviations

ILD: interstitial lung disease
SARD-ILD: ILD associated with systemic autoimmune rheumatic diseases
KL-6: Krebs von den Lungen-6
IL-6: interleukin-6
FER: ferritin
SMRP: soluble mesothelin-related peptide
PFT: pulmonary function tests
HRCT: high-resolution computed tomography
IPF: idiopathic pulmonary fibrosis
DM: dermatomyositis
SSc: systemic sclerosis
RA: rheumatoid arthritis
UK-BILD: UK Biomarkers in Interstitial Lung Disease; pSS primary Sjogren syndrome
SLE: systemic lupus erythematous SLE
MCTD: mixed connective tissue disease
PM: polymyositis
UCTD: undifferentiated connective tissue disease
UIP: usual interstitial pneumonia
NSIP: non-specific interstitial pneumonia
AUROC: areas under the receiver operating characteristic

## References

[1] Senécal JL, Chartier S, Rothfield N (1995) Hypergammaglobulinemic purpura in systemic autoimmune rheumatic diseases: predictive value of anti-Ro(SSA) and anti-La(SSB) antibodies and treatment with indomethacin and hydroxychloroquine. J Rheumatol 22:868–875. 8587074

[2] Guthridge JM, Wagner CA, James JA (2022) The promise of precision medicine in rheumatology. Nat Med 28:1363–1371. doi: 10.1038/s41591-022-01880-6 35788174 PMC9513842

[3] Vij R, Strek ME (2013) Diagnosis and treatment of connective tissue disease-associated interstitial lung disease. Chest 143:814–824. doi: 10.1378/chest.12-0741 23460159 PMC3590889

[4] Hannah J, Rodziewicz M, Mehta P, et al (2024) The diagnosis and management of systemic autoimmune rheumatic disease-related interstitial lung disease: British Society for Rheumatology guideline scope. Rheumatol Adv Pract 8:rkae056. doi: 10.1093/rap/rkae056 38765189 PMC11101284

[5] Mathai SC, Danoff SK (2016) Management of interstitial lung disease associated with connective tissue disease. BMJ 352:h6819. doi: 10.1136/bmj.h6819 26912511 PMC6887350

[6] Walsh SLF, Lederer DJ, Ryerson CJ, et al (2019) Diagnostic Likelihood Thresholds That Define a Working Diagnosis of Idiopathic Pulmonary Fibrosis. Am J Respir Crit Care Med 200:1146–1153. doi: 10.1164/rccm.201903-0493OC 31241357

[7] Ishikawa N, Hattori N, Yokoyama A, Kohno N (2012) Utility of KL-6/MUC1 in the clinical management of interstitial lung diseases. Respir Investig 50:3–13. doi: 10.1016/j.resinv.2012.02.001 22554854

[8] Lin L, Zhao Y, Li Z, et al (2022) Expression of S100A9 and KL-6 in common interstitial lung diseases. Medicine (Baltimore) 101:e29198. doi: 10.1097/MD.0000000000029198 35512076 PMC9276110

[9] Jiang D, Xiao H, Dong R, et al (2022) Krebs von den Lungen-6 levels in untreated idiopathic pulmonary fibrosis. Clin Respir J 16:234–243. doi: 10.1111/crj.13475 35081277 PMC9060088

[10] S S-D, X M, I O, et al (2022) YKL-40 and KL-6 Levels in Serum and Sputum of Patients Diagnosed With Hypersensitivity Pneumonitis. The journal of allergy and clinical immunology In practice 10:. doi: 10.1016/j.jaip.2022.06.031 35788062

[11] Lanzarone N, Gentili F, Alonzi V, et al (2020) Bronchoalveolar lavage and serum KL-6 concentrations in chronic hypersensitivity pneumonitis: correlations with radiological and immunological features. Intern Emerg Med. doi: 10.1007/s11739-020-02281-8 32078140

[12] Fu H, Zheng Z, Zhang Z, et al (2023) Prediction of progressive pulmonary fibrosis in patients with anti-synthetase syndrome-associated interstitial lung disease. Clin Rheumatol 42:1917–1929. doi: 10.1007/s10067-023-06570-3 36929316 PMC10266998

[13] Komai T, Iwasaki Y, Tsuchida Y, et al (2023) Efficacy and safety of plasma exchange in interstitial lung diseases with anti-melanoma differentiation-associated 5 gene antibody positive clinically amyopathic dermatomyositis. Scand J Rheumatol 52:77–83. doi: 10.1080/03009742.2021.1995984 34895028

[14] Zhu Y, Wang L, Sun Y, et al (2022) Serum Krebs von den Lungen-6 concentrations reflect severity of anti-melanoma differentiation-associated protein 5 antibody positive dermatomyositis associated interstitial lung disease. Clin Exp Rheumatol 40:292–297. doi: 10.55563/clinexprheumatol/zmn18h 34874831

[15] d’Alessandro M, Bellisai F, Bergantini L, et al (2021) Prognostic role of KL-6 in SSc-ILD patients with pleuroparenchymal fibroelastosis. Eur J Clin Invest e13543. doi: 10.1111/eci.13543 33759179

[16] Li H, Zhang X, Yu L, et al (2022) Comparing clinical characteristics of systemic sclerosis with or without interstitial lung disease: A cross-sectional study from a single center of the Chinese Rheumatism Data Center. Front Med (Lausanne) 9:1061738. doi: 10.3389/fmed.2022.1061738 36561716 PMC9763297

[17] Yokoyama K, Mitoma H, Kawano S, et al (2022) CEACAM 1, 3, 5 and 6 -positive classical monocytes correlate with interstitial lung disease in early systemic sclerosis. Front Immunol 13:1016914. doi: 10.3389/fimmu.2022.1016914 36341379 PMC9632165

[18] Kuwana M, Shirai Y, Takeuchi T (2016) Elevated Serum Krebs von den Lungen-6 in Early Disease Predicts Subsequent Deterioration of Pulmonary Function in Patients with Systemic Sclerosis and Interstitial Lung Disease. J Rheumatol 43:1825–1831. doi: 10.3899/jrheum.160339 27481907

[19] Weng L, Chen Y, Liang T, et al (2022) Biomarkers of interstitial lung disease associated with primary Sjögren’s syndrome. Eur J Med Res 27:199. 10.1186/s40001-022-00828-3. 36217184 PMC9549683

[20] Chiu Y-H, Chu C-C, Lu C-C, et al (2022) KL-6 as a Biomarker of Interstitial Lung Disease Development in Patients with Sjögren Syndrome: A Retrospective Case-Control Study. J Inflamm Res 15:2255–2262. 10.2147/JIR.S352085. 35422651 PMC9005069

[21] Makino H, Kotani T, Hata K, et al (2022) Prognostic factors affecting respiratory-related death in patients with rheumatoid arthritis complicated by interstitial lung disease: An ANSWER cohort study. Mod Rheumatol roac 115. 10.1093/mr/roac115. 36112486

[22] Qin Y, Wang Y, Meng F, et al (2022) Identification of biomarkers by machine learning classifiers to assist diagnose rheumatoid arthritis-associated interstitial lung disease. Arthritis Res Ther 24:115. doi: 10.1186/s13075-022-02800-2 35590341 PMC9118651

[23] Conticini E, d’Alessandro M, Bergantini L, et al (2022) KL-6 in ANCA-Associated Vasculitis Patients with and without ILD: A Machine Learning Approach. Biology 11:94. doi: 10.3390/biology11010094 35053092 PMC8772774

[24] Lee JS, Lee EY, Ha Y-J, et al (2019) Serum KL-6 levels reflect the severity of interstitial lung disease associated with connective tissue disease. Arthritis Res Ther 21:58. doi: 10.1186/s13075-019-1835-9 30764869 PMC6376648

[25] Recalcati S, Invernizzi P, Arosio P, Cairo G (2008) New functions for an iron storage protein: the role of ferritin in immunity and autoimmunity. J Autoimmun 30:84–89. doi: 10.1016/j.jaut.2007.11.003 18191543

[26] Gono T, Kawaguchi Y, Satoh T, et al (2010) Clinical manifestation and prognostic factor in anti-melanoma differentiation-associated gene 5 antibody-associated interstitial lung disease as a complication of dermatomyositis. Rheumatology (Oxford) 49:1713–1719. doi: 10.1093/rheumatology/keq149 20498012

[27] Shirai T, Machiyama T, Sato H, et al (2023) Intensive induction therapy combining tofacitinib, rituximab and plasma exchange in severe anti-melanoma differentiation-associatedprotein-5 antibody-positive dermatomyositis. Clin Exp Rheumatol 41:291–300. doi: 10.55563/clinexprheumatol/8kulbf 36700661

[28] Mimori T, Nakashima R, Hosono Y (2012) Interstitial lung disease in myositis: clinical subsets, biomarkers, and treatment. Curr Rheumatol Rep 14:264–274. doi: 10.1007/s11926-012-0246-6 22367479

[29] Rump A, Morikawa Y, Tanaka M, et al (2004) Binding of ovarian cancer antigen CA125/MUC16 to mesothelin mediates cell adhesion. J Biol Chem 279:9190–9198. doi: 10.1074/jbc.M312372200 14676194

[30] Aringer M, Costenbader K, Daikh D, et al (2019) 2019 European League Against Rheumatism/American College of Rheumatology Classification Criteria for Systemic Lupus Erythematosus. Arthritis Rheumatol 71:1400–1412. doi: 10.1002/art.40930 31385462 PMC6827566

[31] Aletaha D, Neogi T, Silman AJ, et al (2010) 2010 rheumatoid arthritis classification criteria: an American College of Rheumatology/European League Against Rheumatism collaborative initiative. Ann Rheum Dis 69:1580–1588. doi: 10.1136/ard.2010.138461 20699241

[32] Shiboski CH, Shiboski SC, Seror R, et al (2017) 2016 American College of Rheumatology/European League Against Rheumatism Classification Criteria for Primary Sjögren’s Syndrome: A Consensus and Data-Driven Methodology Involving Three International Patient Cohorts. Arthritis Rheumatol 69:35–45. 10.1002/art.39859. 27785888 PMC5650478

[33] Lundberg IE, Tjärnlund A, Bottai M, et al (2017) 2017 European League Against Rheumatism/American College of Rheumatology classification criteria for adult and juvenile idiopathic inflammatory myopathies and their major subgroups. Ann Rheum Dis 76:1955–1964. doi: 10.1136/annrheumdis-2017-211468 29079590 PMC5736307

[34] van den Hoogen F, Khanna D, Fransen J, et al (2013) 2013 classification criteria for systemic sclerosis: an American college of rheumatology/European league against rheumatism collaborative initiative. Ann Rheum Dis 72:1747–1755. doi: 10.1136/annrheumdis-2013-204424 24092682

[35] (2022) Disease MCTD—ERN ReCONNET | European Reference Network on Rare and Complex Connective Tissue and Musculoskeletal Diseases. https://reconnet.ern-net.eu/disease-mctd/. Accessed 3 Sep 2024. 10.55563/clinexprheumatol/d2qz38 35349419

[36] Mosca M, Neri R, Bombardieri S (1999) Undifferentiated connective tissue diseases (UCTD): a review of the literature and a proposal for preliminary classification criteria. Clin Exp Rheumatol 17:615–620. 10544849

[37] Connors GR, Christopher-Stine L, Oddis CV, Danoff SK (2010) Interstitial lung disease associated with the idiopathic inflammatory myopathies: what progress has been made in the past 35 years? Chest 138:1464–1474. doi: 10.1378/chest.10-0180 21138882

[38] Raghu G, Collard HR, Egan JJ, et al (2011) An official ATS/ERS/JRS/ALAT statement: idiopathic pulmonary fibrosis: evidence-based guidelines for diagnosis and management. Am J Respir Crit Care Med 183:788–824. doi: 10.1164/rccm.2009-040GL 21471066 PMC5450933

[39] Bergantini L, Bargagli E, d’Alessandro M, et al (2021) Prognostic bioindicators in severe COVID-19 patients. Cytokine 141:155455. doi: 10.1016/j.cyto.2021.155455 33548798 PMC7843114

[40] Nakajima H, Harigai M, Hara M, et al (2000) KL-6 as a novel serum marker for interstitial pneumonia associated with collagen diseases. J Rheumatol 27:1164–1170. 10813282

[41] Cjw S, Rk H, C D, et al (2021) Serum markers of pulmonary epithelial damage in systemic sclerosis-associated interstitial lung disease and disease progression. Respirology (Carlton, Vic) 26:. 10.1111/resp.13988. 33336433

[42] Huang Y, Ma S-F, Oldham JM, et al (2024) Machine Learning of Plasma Proteomics Classifies Diagnosis of Interstitial Lung Disease. Am J Respir Crit Care Med 210:444–454. doi: 10.1164/rccm.202309-1692OC 38422478 PMC11351805

[43] Conticini E, d’Alessandro M, Cameli P, et al (2023) Prevalence of myositis specific and associated antibodies in a cohort of patients affected by idiopathic NSIP and no hint of inflammatory myopathies. Immunol Res. 10.1007/s12026-023-09387-z. PMC10517890 37133680

