# Supplementary material for: Panel of serum biomarkers for differential diagnosis of idiopathic interstitial lung disease and interstitial lung disease-secondary to systemic autoimmune rheumatic disease
Source: PLoS One. 2024 Oct 3;19(10):e0311357. doi: 10.1371/journal.pone.0311357 (PMC11449321; doi:10.1371/journal.pone.0311357)
Supplement: S1 Table — The statistically significant variables was marked in bold (p values columns). (DOCX) [file pone.0311357.s001.docx]

Table S1. Binomial logistic regression was performed to understand the effect of demographic (gender, age, ethnicity and smoking history) and immunological (SMRP, FER, KL-6 and IL-6) features on the diagnosis of idiopathic ILD and SARD-ILD. The statistically significant variables was marked in bold (p values columns).

| Model Coefficients - SARD-ILD vs Idiopathic ILD | | | | | | | | | | | | | | | |
| --- | --- | --- | --- | --- | --- | --- | --- | --- | --- | --- | --- | --- | --- | --- | --- |
|  | | | | | | | | | | | | **95% Confidence Interval** | | | |
| **Predictor** | | **Estimate** | | **SE** | | **Z** | | **p** | | **Odds ratio** | | **Lower** | | **Upper** | |
| Intercept |  | 6.74129 |  | 0.65793 |  | 10.24626 |  | < .0001 |  | 846.650 |  | 233.172 |  | 3074.197 |  |
| SMRP nmol/L |  | -0.05466 |  | 0.11492 |  | -0.47568 |  | 0.6343 |  | 0.947 |  | 0.756 |  | 1.186 |  |
| FER ng/mL |  | -0.00210 |  | 7.02e-4 |  | -2.98777 |  | **0.0028** |  | 0.998 |  | 0.997 |  | 0.999 |  |
| IL6 pg/mL |  | -9.36e−6 |  | 3.50e-4 |  | -0.02677 |  | 0.9786 |  | 1.000 |  | 0.999 |  | 1.001 |  |
| KL6 U/mL |  | -1.38e−4 |  | 6.53e-5 |  | -2.11542 |  | **0.0344** |  | 1.000 |  | 1.000 |  | 1.000 |  |
| age |  | -0.07984 |  | 0.00879 |  | -9.08076 |  | **< .0001** |  | 0.923 |  | 0.907 |  | 0.939 |  |
| gender: |  |  |  |  |  |  |  |  |  |  |  |  |  |  |  |
| Male – Female |  | -1.52468 |  | 0.16292 |  | -9.35841 |  | **< .0001** |  | 0.218 |  | 0.158 |  | 0.300 |  |
| smoking history: |  |  |  |  |  |  |  |  |  |  |  |  |  |  |  |
| Ex – Never |  | -0.22199 |  | 0.16542 |  | -1.34194 |  | 0.1796 |  | 0.801 |  | 0.579 |  | 1.108 |  |
| Current – Never |  | 0.21046 |  | 0.46041 |  | 0.45713 |  | 0.6476 |  | 1.234 |  | 0.501 |  | 3.043 |  |
| ethnicity: |  |  |  |  |  |  |  |  |  |  |  |  |  |  |  |
| Asian – Caucasian |  | 2.03913 |  | 0.49089 |  | 4.15397 |  | **< .0001** |  | 7.684 |  | 2.936 |  | 20.111 |  |
| African – Caucasian |  | 14.63085 |  | 544.99641 |  | 0.02685 |  | 0.9786 |  | 2.26e+6 |  | 0.000 |  | Inf |  |
| Afro-Carribean – Caucasian |  | 3.02710 |  | 1.07911 |  | 2.80518 |  | **0.0050** |  | 20.637 |  | 2.490 |  | 171.078 |  |
| Mixed - specify – Caucasian |  | 15.07958 |  | 777.22351 |  | 0.01940 |  | 0.9845 |  | 3.54e+6 |  | 0.000 |  | Inf |  |
| Others - specify – Caucasian |  | 14.85370 |  | 780.95287 |  | 0.01902 |  | 0.9848 |  | 2.82e+6 |  | 0.000 |  | Inf |  |
| Mixed - specify – Caucasian |  | -15.95853 |  | 2399.54474 |  | -0.00665 |  | 0.9947 |  | 1.17e-7 |  | 0.000 |  | Inf |  |
| Note. Estimates represent the log odds of "idiopathic ILD" vs. "SARD-ILD" | | | | | | | | | | | | | | | |
|  | | | | | | | | | | | | | | | |
