# Supplementary material for: Panel of serum biomarkers for differential diagnosis of idiopathic interstitial lung disease and interstitial lung disease-secondary to systemic autoimmune rheumatic disease
Source: PLoS One. 2024 Oct 3;19(10):e0311357. doi: 10.1371/journal.pone.0311357 (PMC11449321; doi:10.1371/journal.pone.0311357)

Figure s1. The scree plot method for Kaiser-Guttman’s rule to conduct supervised Principal Component Analysis in an exploratory approach for identifying trends in immunological (KL-6, IL-6, SMRP, FER) and demographic (age) features by 2D representation of the multi-dimensional data set.


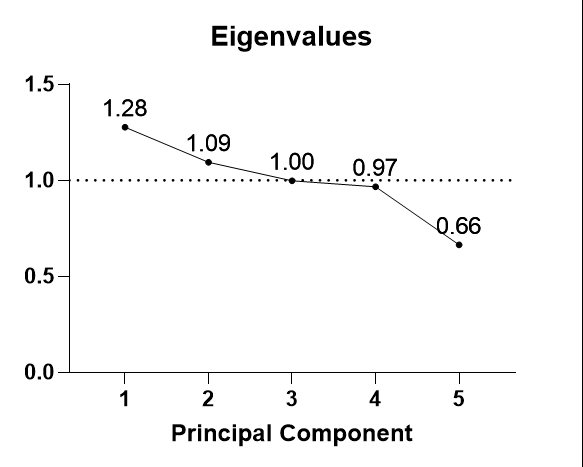

Supplement: S1 Fig — (DOCX) [file pone.0311357.s002.docx]
