# Supplementary material for: Panel of serum biomarkers for differential diagnosis of idiopathic interstitial lung disease and interstitial lung disease-secondary to systemic autoimmune rheumatic disease
Source: PLoS One. 2024 Oct 3;19(10):e0311357. doi: 10.1371/journal.pone.0311357 (PMC11449321; doi:10.1371/journal.pone.0311357)
Supplement: Attachment — Submitted filename: point by point reply UKBILD-Plos One.docx [file pone.0311357.s003.docx]

Dear Editor,

The authors would like to thank you and appreciate the Reviewer for the constructive comments. We also thank the Reviewer for the effort and time put into the review of the manuscript. The comments are encouraging, and the Reviewer appears to share our judgement that this study and its results are important and worth publication. Each comment has been carefully considered point by point and responded to. Please see below our detailed response to the comments raised, which you can find in italic. Changes provided in the revised manuscript are in red. The manuscript has been revised according to the Reviewer’s suggestions and has been significantly improved. We are hoping that its final version deserves publication in “Plos One”.

Reviewer #1: The is the biomarker study using the large scale of ILD cases. In addition to well-known KL-6, IL-6, and ferritin, a novel marker, SMRP, is investigated as well. Although this is the hot topics of ILD field, I have following concerns.

Major points

[1] The rationale of the study does not seem clear.

Why should the authors use biomarkers to further distinguish between primary and secondary conditions that are already clinically and clearly distinct? It is not clear what is unresolved and why this study is needed.

Thanks for giving us the chance to clarify such a crucial aspect of our study. In the common clinical practice, clearly distinguishing between primary and secondary ILD is not always easy. For instance, it is not so uncommon to have patients who were previously diagnosed with idiopathic NSIP and then display a positivity of any MSA. This is, according to us, a major point, because while idiopathic ILD may benefit from antifibrotic agents, secondary ones may require a prompt immunosuppressive treatment. Indeed, a multidisciplinary assessment is highly recommended by international guidelines for the diagnostic pathway of ILD, including at least pulmonologists, radiologists, rheumatologists, in order to optimise the diagnostic accuracy and guarantee the earliest and more proper therapeutic proposal. It is worthy to consider that, despite this recommendation, a significant percentage of ILD patients still receives a “working diagnosis”, since clinical symptoms and immunological assessment may not always be sufficient for a confident diagnosis and potentially invasive samplings (such as cryobiopsy or lung surgical biopsy) may not be suitable due to the frailty of clinical conditions (Am J Respir Crit Care Med. 2019 Nov 1;200(9):1146-1153. doi: 10.1164/rccm.201903-0493OC.). For this reason, the validation and discovery of non-invasive and reproducible biomarkers that may help to improve our diagnostic accuracy is highly needed.

In this setting, in order to validate our findings and our proposed biomarkers, we needed a cohort of patients in whom a definite diagnosis was already performed.

We added such finding in the introduction section of revised manuscript as follows:

“A multidisciplinary assessment is highly recommended by international guidelines for the diagnostic pathway of ILD, including at least pulmonologists, radiologists, rheumatologists, in order to optimise the diagnostic accuracy and guarantee the earliest and more proper therapeutic proposal. Despite this recommendation, a significant percentage of ILD patients still receives a “working diagnosis”, since clinical symptoms and immunological assessment may not always be sufficient for a confident diagnosis and potentially invasive samplings (such as cryobiopsy or lung surgical biopsy) may not be suitable due to the frailty of clinical conditions (Am J Respir Crit Care Med. 2019 Nov 1;200(9):1146-1153. doi: 10.1164/rccm.201903-0493OC.)”

[2] Abstract and Results

1) “Primary and secondary-ILD”

Define what is “primary ILD” and “secondary ILD”.

Thanks for evidencing this mistake. “Primary” and “secondary” ILD were changed into “idiopathic” and “SARD”: we hope that the manuscript could look now more homogeneous in terminology

2) “ARD”

Systemic autoimmune rheumatic diseases (SARDs) may be better to use.

The definition has been modified accordingly

3) “idiopathic ILD”, “idiopathic UIP”, “idiopathic NSIP”, “ARD-NSIP”, “ARD-UIP”

What kind of guidelines have used these terms? Proper terminology and definitions are needed.

Thanks for raising this point. pSS, RA, SLE, MCTD, SSc and IIM were diagnosed according to currently available classification criteria (see ref. 24-27), while idiopathic ILD according to American Thoracic Society/European Respiratory Society (ATS/ERS) guidelines (ref. 28).

[3] Introduction

Many of the references on the role of KL-6 are published around 2022, but the authors should cite the original paper on the topics. In researching the field, the authors do not appear to have a good grasp of previous research. Reviewing the references once again. Here are the samples.

1) Instead of reference #3, the following paper may help the authors in literature search.

Ishikawa N, Hattori N, Yokoyama A, Kohno N. Respir. Investig. 2012, 50, 3-13.

2) KL-6 was most studied in SSc-ILD. KL-6 was shown to have a prognostic role on the following paper.

Kuwana M, Shirai Y, Takeuchi T. J Rheumatol. 2016 Oct;43(10):1825-1831.

Thank you for the suggestion, we modified it accordingly.

[4] Discussion

The conclusion part is too long and not coherent.

Thanks for the suggestion: the conclusion has been shortened to a few bullet points which are more useful for the comprehension of the manuscript.

Minor points

[5] Introduction

Ferritin, IL-6, and KL-6 are familiar in the field of ILD. However, SMRP is not.

Explain the reason the authors picked up and investigate it among so many biomarker candidates.

Thanks for raising this important point: as correctly stated, no study has to date evaluated the role of SMRP in ILD. Nevertheless, recent evidence highlights the role of mesothelin (MSLN which binds cancer antigen CA-125 also known as MUC16) in pulmonary fibrosis, suggesting that MSLN is involved in cell adhesion [Rump et al., 2004]. The literature also reports a role of MUC16 in the development and progression of IPF through the TGF-β1 canonical pathway. In this regard, taking action from these preliminary findings, we decided to assess whether SMRP, aside from “classical” biomarkers, could give interesting insights in this field. Such a statement has been added in “Introduction” section.

[6] Methods

1) The authors have not mentioned the definition of SSc, MCTD, and UCTD.

Thank you. The classification criteria of the abovementioned criteria has been added, accordingly.

New references were added in the revised manuscript:

SSC: [24122180](https://pubmed.ncbi.nlm.nih.gov/24122180)

MCTD: R. Kasukawa, T. Too, S. Miyawaki, H. Yoshida, K. Tanimoto, M. Nobunaga, *et al.* Preliminary diagnostic criteria for classification of mixed connective tissue disease

UCTD: “Due to the lack of validated diagnostic or classification criteria, we employed the preliminary ones relased in 1999 (10544849)

ASS: 21138882

2) Mention the definition of “pulmonary hypertension” listed in the Table 1.

Thanks for your comment. According to your advice, we have added the definition of pulmonary hypertension as defined by international guidelines, that we apply for our patients in the multidisciplinary evaluation according to our Centre protocol.

3) The results of logistic regression analysis and multivariate analysis were described in the discussion part. Explain them in the methods part.

Thank you for the comment. We moved the results of logistic regression analysis and multivariate analysis in the results section.

4) Who examined the HRCT findings?

Thanks for your comment that helped us to further clarify this aspect for the readers. In the Centres included in the study, all patients with a clinical suspect or diagnosis of ILD undergo a specific diagnostic pathway, including a multidisciplinary discussion to optimise the diagnostic accuracy, according to international guidelines. Therefore, HRCT images are performed and reviewed by radiologists with a specific expertise on this field, that are part of the multidisciplinary group.

To better clarify this point, we have added a specific sentence in the Methods’ section.

[7] Figure 1a

We can see 0 and 1 groups. Labeling is required.

Thanks for the comment. We modified it accordingly. Moreover, we replaced “SARD-ILD” instead of “ARD-ILD” as suggested above.

[8] Figure 2a

Why did the authors bring IL-6 into the analysis? IL-6 was not shown to distinguish the two groups significantly in Figure 1?

Thank you for the comment. We performed the unsupervised machine learning analysis with variable importance plot to construct a model selecting variables to make accurate predictions. In figure 2a we reported all variables, though not statistically significant in the comparative analysis, to demonstrate that IL-6 did not affect the diagnosis SARD-ILD vs Idiopathic ILD. Here is reported the variable importance plot without IL-6 concentrations.

[9] Figure 2a and 4

The letter size of the labeling is too small to see.

Thank you for the comment. We modified the figures accordingly.

Reviewer #2: This paper investigates the utility of KL-6, ferritin, IL-6, and SMRP (soluble mesothelin-related peptide) as biomarkers in distinguishing idiopathic interstitial lung disease (ILD) from ILD associated with autoimmune rheumatic diseases. It proposes a diagnostic model that incorporates clinical background information. Although the study is insightful, the choice of biomarkers, particularly IL-6 and SMRP, appears abrupt and lacks sufficient rationale. The introduction provides a rich explanation of KL-6, but the discussion of ferritin, IL-6, and SMRP in relation to ILD, supported by previous studies, is insufficient. A more robust and logical explanation for selecting these biomarkers is needed. Additionally, the quality of the figures, including resolution and text size, needs improvement to meet the standards of a scholarly article. While the discussion highlights the strengths of the study, it falls short of comparing the findings with previous research, especially concerning the use of machine learning in ILD differentiation and modeling.

Overall, the study's design, the results presented (including the quality of figures), the logical structure of the discussion, and the alignment between the content of the paper and the conclusions emphasized in the abstract are lacking in maturity and refinement. Below are the identified issues:

Major Comments:

1. As mentioned in the overall assessment, the selection of IL-6 and SMRP from the myriad of inflammatory cytokines appears abrupt. The introduction provides a detailed explanation of KL-6, but the discussion of ferritin (FER), IL-6, and SMRP, supported by previous studies, in relation to ILD is insufficient. A clear rationale for selecting IL-6 and SMRP from the numerous ILD-related biomarkers is required.

Thanks for raising this important point: as correctly stated, no study has to date evaluated the role of SMRP in ILD. Nevertheless, recent evidence highlights the role of mesothelin (MSLN which binds cancer antigen CA-125 also known as MUC16) in pulmonary fibrosis, suggesting that MSLN is involved in cell adhesion [Rump et al., 2004]. The literature also reports a role of MUC16 in the development and progression of IPF through the TGF-β1 canonical pathway. In this regard, taking action from these preliminary findings, we decided to assess whether SMRP, aside from “classical” biomarkers, could give interesting insights in this field. Such a statement has been added in “Introduction” section.

Interleukin-6 (IL-6) is a pleiotropic cytokine involved in the physiology of virtually every organ system. Controlling IL-6 activity is potentially an effective approach in the treatment of various autoimmune and inflammatory diseases. The recent introduction of tocilizumab, a humanised monoclonal antibody targeting IL-6R, is further evidence of the role of IL-6 in the pathogenesis of RA. Baricitinib is a JAK inhibitor that blocks intracellular signalling pathways of inflammatory cytokines recommended for Rheumatoid arthritis (RA) patients not responding to initial treatment. Baricitinib was demonstrated to be a safe immune modulator that reduces the concentrations of biomarkers of lung fibrosis and inflammation in RA patients, including a subgroup with interstitial lung involvement.

As a result, we included IL-6 and SMRP concentrations in different SARD-ILD compared with idiopathic ILD.

2. From a clinical application perspective, while specialized tests like IL-6 and SMRP are valuable, the study should also consider practical biomarkers like SP-D and SP-A, which are used in clinical settings alongside KL-6.

Thank you for the suggestion. We selected four markers detectable at the same time using a few microliters (about 150uL) of serum through the chemiluminescence method using Fujirebio reagents. We included KL-6, ferritin and IL-6 as valuable markers in the contest of idiopathic interstitial lung diseases and in a few types of SARD-ILD (e.g. RA).

To date no study has evaluated the role of SMRP in ILD. Nevertheless, recent evidence highlights the role of mesothelin (MSLN which binds cancer antigen CA-125 also known as MUC16) in pulmonary fibrosis, suggesting that MSLN is involved in cell adhesion. An epithelial cell's apical surface is covered with transmembrane mucins, which are large glycoproteins. Cell surface mucins are characterized by non-covalent sodium dodecyl sulfate-labile bonds holding dimerizations of two dissimilar subunits together (α and β chains). It is highly glycosylated and entirely extracellular. High glycosylation levels in this extracellular domain contribute to barrier formation in addition to protecting the protein backbone from proteolytic attacks by hosts. In addition to the putative phosphorylation sites present in all transmembrane mucins intracellular tails, these tails differ in sequence and length and do not contain conserved domains. It is nevertheless believed that transmembrane mucins contribute significantly to cellular proliferation, apoptosis, and epithelial to mesenchymal transition processes, in accordance with IPF observations. According to the above information, the lung is predominantly composed of MUC1, MUC4, and MUC16 TM mucins.

3. In the Methods section, if there is a classification criterion for ARD, it should be included. Is there a specific reason for using ARD instead of connective tissue disease (CTD)? The basis for selecting various symptoms and examination findings, such as elevated CK, as ARD signs should be explained with reference to literature.

Thanks for raising this important point. All classification criteria were added (unfortunately, some of them were not mentioned in the first version of the manuscript) and can now be found among the references. In terms of inclusion criteria, we reckon that the choice of including not only CTD but ARD “in general” can not easily understandable and deserves some clarifications: first, the occurrence of ILD is not limited to CTD, but has been reported in up to 10% of patients with RA and its precocious diagnosis and management remain an authentic dilemma. Secondly, the most common pattern of ILD in RA is UIP, which, in turn, is usually present only in SSc among CTDs, which generally have a NSIP pattern. At the same time, the pathological mechanisms of RA, driven also by IL-6 (which is less prominent in SSc, as proved by the uncertain efficacy of TCZ in this condition), could potentially aid in evaluating differences and similarities of ILD development in such diseases. Same when comparing RA with IIM and other CTDs, in which the role of IL-6 is classically considered less important, while other agents (e.g. B cells, IFN) carry a crucial pathogenetic role. Therefore, if excluding such an important and prevalent condition like RA would have been a strong limitation of our study, its inclusion may hopefully strongly corroborate our findings.

4. The Methods section lacks citations related to the classification of MCTD and SSc. Anti-synthetase syndrome and UCTD are mentioned in the Results, but their classification criteria should be referenced in the Methods section.

Thank you: all missing criteria were included.

5. The definitions and classifications of chest HRCT findings in ILD should be organized and included in the Methods section, not the Results.

Thank you for the comment. We reorganised the methods and results sections accordingly.

6. The resolution of all figures is poor, and the text is faint and small, making them difficult to read.

Thank you for the comment. We increased the resolution of all figures and included them in the revised manuscript as you suggested.

7. In Figure 1a, it is unclear whether the vertical axis values (0 or 1) correspond to idiopathic ILD or ARD-ILD.

Thank you for the comment. We clarified the axis values as follow:

8. Detailed descriptions of the statistical methods used in Principal Component Analysis are necessary. Was the analysis conducted according to Kaiser-Guttman’s rule using the scree plot method? A scree plot should be added as a supplementary figure. How were the absolute variables selected?

Thank you for the comment. We conducted PCA analysis using the Kaiser-Guttman rule (the number of factors is equal to the number of factors with eigenvalues greater than 1.0) with scree plot method in the revised manuscript. We added the scree plot as a supplementary figure. We selected the continuous variables for PCA analysis, we reported in the methods section the following sentence: “Supervised Principal Component Analysis using Kaiser-Guttman rule was performed in an exploratory approach to identify trends in immunological (KL-6, IL-6, SMRP, FER) and demographic (age) features by 2D representation of the multi-dimensional data set.”

9. In Table 2, why does the number of diseases compared vary for each serum biomarker? What does "W" stand for?

Thank you for the comment. Patients were further stratified according to HRCT findings and comparative analysis of serum marker concentrations were performed within and between the following groups: the idiopathic ILD group included probable fibrotic NSIP on HRCT (IPF confirmed at multidisciplinary discussion, >65 years old, without UIP confirmation at lung biopsy), definite UIP and definite fibrotic NSIP (UIP confirmed at lung biopsy). For SARD patients, those with RA and SSc displaying a UIP pattern (SARD-UIP) were considered separately from those with NSIP (SARD-NSIP).

The “W” stands for weight. A weight in statistical terms is defined as a coefficient assigned to a number in a computation, for example when determining an average, to make the number's effect on the computation reflect its importance.

10. In Figure 4, the diseases compared are unclear. It seems that only statistically significant differences are presented, but the rationale is not provided, making it difficult to understand. The figure legends are also insufficient.

Thank you for the comment. We divided patients with SARD according to the specific diagnosis and serum markers, and were compared within and between groups, as well as with the idiopathic ILD group. Figure 4 reported the box plots of each serum marker in the disease groups. The tables below each box plot reported the statistically significant differences of serum marker concentrations between disease groups. We modified the figure legend as follows:

“Figure 4. Box plots reported serum marker concentrations in idiopathic interstitial lung disease (ILD) versus SARD-ILD subgroups: rheumatoid arthritis (RA), systemic lupus erythematous (SLE), mixed connective tissue disease (MCTD), polymyositis (PM), dermatomyositis (DM), systemic sclerosis (SSc), undifferentiated connective tissue disease (UCTD), connective tissue disease (CTD). The statistically significant differences of each serum marker concentration between the disease groups were reported in the tables below the box plots.”

11. The discussion focuses on emphasizing the strengths of the study's findings, but lacks a comparison with previous research, especially regarding ILD differentiation using machine learning and related models.

Thank you for the suggestion. We totally agree with the reviewer and we added the recent literature data about the using of machine learning and related models in ILD differentiation integrating new references as follows:

“Identification of biomarkers by machine learning classifiers to assist diagnose RA-ILD has been proposed by Qin et al (ref doi:10.1186/s13075-022-02800-2). KL-6 concentration, D-dimer, and tumor markers greatly aided RA-ILD identification. Machine learning algorithms combined with traditional biostatistical analysis could be helpful to diagnose RA-ILD patients and identify biomarkers potentially associated with the disease. Recently, Huang et al (ref DOI: [10.1164/rccm.202309-1692OC](https://doi.org/10.1164/rccm.202309-1692oc) ) proposed multiple machine learning models trained with a large number of proteins involved in the immune pathway consistently distinguished CTD-ILD from IPF in challenging cases and improved clinical decision making.”

12. In the Abstract’s discussion, the focus should be on proposing an "ILD differentiation model including clinical background," but instead, the emphasis seems to be on the utility of serum SMRP levels.

Thank you for the comment. We modified the abstract’s discussion as follows: “Our study proposed an ILD differentiation model including clinical background. In this context, combination of serum markers and clinical data, as seen in our cohort, may lead to a further improvement in diagnostic accuracy for ILD.”.

Minor Comments:

13. In Table 1, the ARD subgroup should be arranged in the order of SSc Limited, SSc Diffuse, UCTD, and CTD (Unknown).

Thank you for the comment. Table 1 reported the clinical and demographic data in the two disease groups: SARD-ILD and idiopathic ILD. In terms of inclusion criteria, we reckon that the choice of including not only CTD but ARD “in general” can not easily be understood and deserves some clarifications, discussed above (comment 3). First, the occurrence of ILD is not limited to CTD, but has been reported in up to 10% of patients with RA and its precocious diagnosis and management remain an authentic dilemma. Secondly, the most common pattern of ILD in RA is UIP, which, in turn, is usually present only in SSc among CTDs, which generally have a NSIP pattern. At the same time, the pathological mechanisms of RA, driven also by IL-6 (which is less prominent in SSc, as proved by the uncertain efficacy of TCZ in this condition), could potentially aid in evaluating differences and similarities of ILD development in such diseases. Same when comparing RA with IIM and other CTDs, in which the role of IL-6 is classically considered less important, while other agents (e.g. B cells, IFN) carry a crucial pathogenetic role. Therefore, if excluding such an important and prevalent condition like RA would have been a strong limitation of our study, its inclusion may hopefully strongly corroborate our findings.

14. In the Binomial logistic regression analysis (Table S1), it would be less confusing for readers if the comparison targets were labeled as idiopathic ILD and ARD-ILD rather than primary and secondary ILD.

Thank you for the comment. We modified accordingly:

“Table S1. Binomial logistic regression was performed to understand the effect of demographic (gender, age, ethnicity and smoking history) and immunological (SMRP, FER, KL-6 and IL-6) features on the diagnosis of idiopathic ILD and SARD-ILD. The statistically significant variables was marked in bold (p values columns).”

“ Note. Estimates represent the log odds of "idiopathic ILD" vs. "SARD-ILD" “
