# Supplementary material for: Panel of serum biomarkers for differential diagnosis of idiopathic interstitial lung disease and interstitial lung disease-secondary to systemic autoimmune rheumatic disease
Source: PLoS One. 2024 Oct 3;19(10):e0311357. doi: 10.1371/journal.pone.0311357 (PMC11449321; doi:10.1371/journal.pone.0311357)
Supplement: Attachment — Submitted filename: point-by-point reply_rev2.docx [file pone.0311357.s004.docx]

Dear Editor,

The authors would like to thank you and appreciate the Reviewer for the constructive comments. We also thank the Reviewer for the effort and time put into the review of the manuscript. The comments are encouraging, and the Reviewer appears to share our judgement that this study and its results are important and worth publication. Each comment has been carefully considered point by point and responded to. Please see below our detailed response to the comments raised, which you can find in italic. Changes provided in the revised manuscript are in red. The manuscript has been revised according to the Reviewer’s suggestions and has been significantly improved. We are hoping that its final version deserves publication in “Plos One”.

Reviewer #2: Comments for Authors

Regarding Major Comment 3, the response provided is insufficient.

The question raised was whether there exists a previously established definition for the term or disease concept of ARD (systemic ARD). For instance, the concept of UCTD has been in use since its proposal by LeRoy et al. (LeRoy EC, Maricq HR, Kahaleh MB. Undifferentiated connective tissue syndromes. Arthritis Rheum. 1980 Jan;23(1):1-9.). Is there an established concept for SARD? If so, a reference should be cited, and the concept should be briefly described. If it is not an established term (i.e., coined by the authors), a brief explanation of the rationale behind using the term "SARD" should be provided.

We are sorry for the unclear answer to your question. Systemic autoimmune rheumatic diseases (SARD) is a term that covers a broad spectrum of clinical conditions of autoimmune aetiology and, in particular, ILD has been reported in association with several SARD, particularly RA, SSc, IIM, CTD and AAV. The term was first proposed in 1995 (Sénecal et al., J Rheumatol) in a cohort of patients displaying anti-SSA/SSB positivity and the most recent, comprehensive, definition was given in a recently published review (Guthridge et al., Nat Med, 2022, PMID 35788174), whose reference has been therefore added to the bibliography of our manuscript. Finally, specifically focusing on lung involvement in rheumatic diseases, the recently published recommendations of the British Society of Rheumatology employ the term SARD for ILD associated with CTD, AAV, IIM, RA etc. (Hannah et al., Rheumatol Adv Pract, 2024)

Regarding Major Comment 9, the response is again insufficient.

If Table 2 presents only statistically significant results, this should be clearly stated in the figure legend. Additionally, “W” should be described as “weight” in the legend.

Thank you for the comment. We included the description of “W” at the top of the column and we improved the caption of table 2 as follows:

“Table 2. Serum markers concentrations in groups of patients stratified according to HRCT findings: the idiopathic ILD group included probable fibrotic NSIP on HRCT (IPF confirmed at multidisciplinary discussion, >65 years old, without UIP confirmation at lung biopsy), definite UIP and definite fibrotic NSIP (UIP confirmed at lung biopsy). For SARD patients, those with RA and SSc displaying a UIP pattern (SARD-UIP) were considered separately from those with NSIP (SARD-NSIP). Abbreviations: SARD, autoimmune rheumatic disease; NSIP, non-specific interstitial pneumonia; UIP, usual interstitial pneumonia; KL-6, krebs von den lungen-6; FER, ferritin; IL-6, interleukin-6; SMRP, soluble mesothelin-related peptide.”

Regarding Minor Comment 13, Table 1 has not been revised as requested.

The authors' response is also unclear. Please arrange the order of the ARD subgroup diseases in Table 1 as follows: SSc Limited, SSc Diffuse, UCTD, etc.

Thank you for the suggestions. We modified the table 1 accordingly.
